# Equivalent Impedance Models for Electrochemical Nanosensor-Based Integrated System Design

**DOI:** 10.3390/s21093259

**Published:** 2021-05-08

**Authors:** Zhongzheng Wang, Aidan Murphy, Alan O’Riordan, Ivan O’Connell

**Affiliations:** Microelectronic Circuits Centre Ireland, T12 R5CP Cork, Ireland; zhongzheng.wang@mcci.ie (Z.W.); aidan.murphy@mcci.ie (A.M.); alan.oriordan@tyndall.ie (A.O.)

**Keywords:** electrochemical sensors model, Randles Model, sensor layout design, model

## Abstract

Models of electrochemical sensors play a critical role for electronic engineers in designing electrochemical nanosensor-based integrated systems and are also widely used in analyzing chemical reactions to model the current, electrical potential, and impedance occurring at the surface of an electrode. However, the use of jargon and the different perspectives of scientists and electronic engineers often result in different viewpoints on principles of electrochemical models, which can impede the effective development of sensor technology. This paper is aimed to fill the knowledge gap between electronic engineers and scientists by providing a review and an analysis of electrochemical models. First, a brief review of the electrochemical sensor mechanism from a scientist’s perspective is presented. Then a general model, which reflects a more realistic situation of nanosensors is proposed from an electronic engineer point of view and a comparison between the Randles Model is given with its application in electrochemical impedance spectroscopy and general sensor design. Finally, with the help of the proposed equivalent model, a cohesive explanation of the scan rate of cyclic voltammetry is discussed. The information of this paper can contribute to enriching the knowledge of electrochemical sensor models for scientists and is also able to guide the electronic engineer on designing next-generation sensor layouts.

## 1. Introduction

The need for point-of-use monitoring in a wide range of applications including healthcare, pharmaceutical production, environmental protection, agriculture and food production is well established [1]. A wide variety of sensing technologies have been developed in recent years to address the challenges associated with both offline and inline continuous monitoring [1,2,3,4]. The electrochemical sensor is being widely adopted as a mainstream sensing option. Electrochemical sensors utilise a chemical reaction at the surface at one of the electrodes, which is dependent on the presence of a target analyte to alter the impedance of the sensor [5,6,7,8,9]. The change in impedance is detected as a change in current when a known potential is applied to the sensor. Today, electrochemical sensors are utilised in a wide range of applications, including DNA detection [10], glucose monitoring [11,12], serotonin levels in brain tissue [13], virus detection [14,15], enzyme reactions [16], and bacteria detection [17]. Compared to other sensor technologies, electrochemical sensors can provide a much higher resolution and faster response times [18], and its sensing system can consume minimal power [19]. Electrochemical sensors can also reduce the cost and simplify the utilisation related to sample labelling techniques [20]. Owing to their excellent performance and low-power characteristic, electrochemical sensors are commonly employed for mobile platforms and implantable platforms [21,22].

With the advent of the Internet of Things (IoT), electrochemical sensors are also being rapidly adopted for edge devices for industrial and residential safety, point-of-care (PoC) diagnostics and medical diagnostics. The global market for electrochemical sensors was valued at USD $2.19 Billion in 2019 and is expected to almost double to USD $11.83 Billion by 2025 [23,24,25,26]. In the context of the current global COVID-19 pandemic, the need for rapid, stable, accurate, low cost, easy to use sensors has never been greater.

With the recent advances in electrochemical sensor technology and their adoption for commercial sensing applications, some of which is driven by the advent of digital glucose meters, there is a need to develop a single electrical equivalent model of the electrochemical sensor. This model needs to reflect the physical attributes of the sensor to the model to truly reflect the multidisciplinary aspect of electrochemical sensors. This paper provides an overview of electrochemical sensor concepts from an electronic engineer perspective, and provides detailed analysis and descriptions of electrochemical sensor models, then highlights the impact of the sensor design on the resultant model, for both scientists and engineers. This will enable a better understanding of the electrochemical sensor from an electrical perspective, resulting in better and more targeted interface electronics. In Section 2, the voltammetric sensor mechanism will be introduced and reviewed. In Section 3, an equivalent impedance model for electrochemical nanosensor-based integrated systems will be proposed and discussed. Section 4 will address the application of the proposed model including electrochemical impedance spectroscopy and sensor design guides. In Section 5, the impact of the individual components of the model will be examined. Finally, the conclusions are presented in Section 6.

## 2. Electrochemical Sensors

### 2.1. Electrochemical Immunosensor

**Figure 1:**
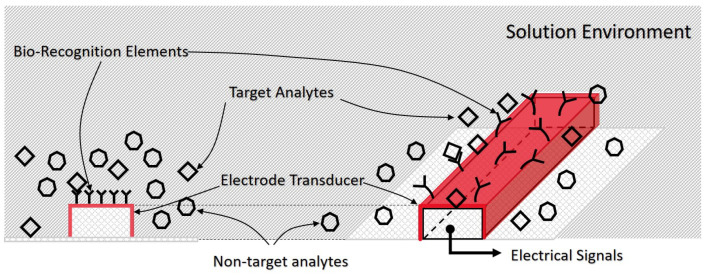
The cross-section view and perspective view of a general electrochemical immunosensor.

A typical electrochemical immunosensor normally composes of four components: the solution (which is the environment in which the sensing occurs), the electrodes (which provides an electrical connection to the solution), the bio-recognition elements (which will only react or bind with the target analyte) and the target analyte (which is the specific element which is being detected and is potentially present in the solution) as is shown in Figure 1. Target analytes can include DNA, tissue, virus, enzymes, bacteria, and other species mentioned in [3]. The bio-recognition elements or sensitive biological elements [27] are typically large protein molecules, which are selective only to a specific target analyte and are largely unaffected by other species or other substances present in the solution. Once the bio-recognition elements interact with an analyte, a change in the electrical characteristics of the sensor occurs, which can be measured and processed by surrounding electrical circuits. Some non-specific binding will also occur arising from the presence of other proteins and cellar materials existing in complex bio-media. The solution environment typically consists of electrolyte, target analytes, and provides the environment for the chemical or biological reactions, and help transport the target analytes to the bio-recognition elements. Besides, the solution also provides an electrical path where electrons move from one electrode to the other.

### 2.2. Voltammetric Sensors

Voltammetric electrochemical sensors are used to study the relationship between an applied potential to an electrochemical cell and the resulting current. The electrochemical sensor is interrogated by sweeping the applied electrical potential across the electrochemical cell from one potential value to another and the resulting current is recorded as a function of applied potential.

The transducer element of voltammetric sensors is the working electrode and the electrons contribute to producing the electrical signal. The principle of the selectivity of a specific analyte is based on the dependence of the measured current and applied potential, and this dependence is a function of the standard potential of the redox couple of interest [28].

**Figure 2:**
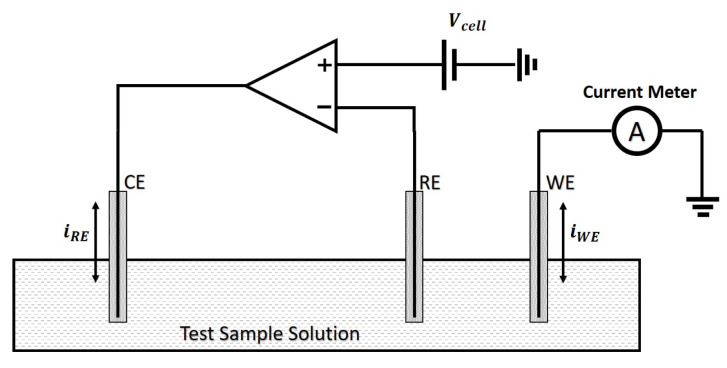
A commonly used configuration of potentiostat for three-electrode amperometric or voltammetric sensors.

In principle, both voltammetric sensors should work with only two electrodes. Nevertheless, in practice, it is difficult to manipulate the potential of an electrode when there is an electrical current passing through it. Therefore, three-electrode voltammetric sensors are introduced to provide more accurate control of the electrical potential at the working electrode, as is shown in Figure 2 [29].

Three-electrode voltammetric sensors have a Working Electrode (WE), a Reference Electrode (RE), and a Counter Electrode (CE). The CE acts as a source/sink of electrons into the test sample solution to enable the electrochemical reaction to occur unimpeded at the WE. Hence the electrons flow from the CE to the WE or vice versa. The RE, which does not have any current flow associated with it and instead senses the electrical potential of the sample solution. In the commonly used potentiostat configuration shown in Figure 2 [30], the amplifier works together with the CE and RE, in a negative feedback loop to ensure that the desired electrical potential is applied to the WE, which is where the electrochemical reaction of interest occurs. By directly sensing the electrical potential of the solution via the RE, any electrical potential drop across the CE is compensated for by the negative feedback loop. As the RE is connected to a high impedance node, the amplifier input, there is no current flow and therefore there is no electrical potential drop across the sample solution RE interface. The introduction of the RE separates the electric current flow path from the sensing path. This will be discussed in more detail in Section 3.

Among the various types of electrochemical sensors, voltammetric based sensors are widely used in medical healthcare [12,31], gas identification and monitoring [32], portable devices [15,21,33], and conducting scientific researches on electrochemical reaction analysis or sensor data acquisition circuits design [30,34,35]. Therefore, a deep understanding of its equivalent electrical model is of great significance [36,37].

## 3. Equivalent Model for Electrochemical Sensor

In order to understand the proposed equivalent model, the conductor-solution interface will be introduced firstly, then a general equivalent model of a voltammetric electrochemical sensor will be proposed. Finally, the widely accepted Randles Model will be presented, and differences between that and the proposed model will be discussed.

### 3.1. Electrode-Solution Interface

**Figure 3:**
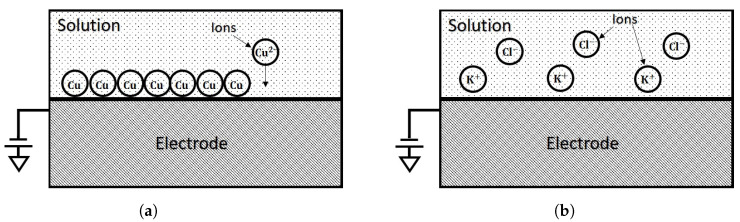
The different types of conductor-solution interfaces of electrodes: (**a**) The conductor-solution interfaces for ideally polarizable electrodes.The charges transfer across the conductor-solution interface; (**b**) The conductor-solution interfaces for non-ideally polarizable electrodes. The charges can not transfer across the conductor-solution interface.

The conductor-solution interface of electrodes where the chemical redox or reduction reactions occur can be classified into two distinct types [38]: (1) Those where the charges can transfer between the electrode surface and solution. This sort of electrodes is also referred to as a non-ideally polarizable electrode, as is shown in Figure 3a, and (2) those where the charges are not permitted to transfer between the electrode surface and solution. This type of electrode is called the ideally polarizable electrode, as is shown in Figure 3b.

#### 3.1.1. Faradaic Process Current

At the interface of non-ideally polarizable electrodes, the electric charges can exchange between the electrode and the solution via ions or electrons directly via the redox reaction, which will result in a current between the solution and electrode. This current, caused by a redox reaction with an applied potential at the electrode, is called the faradaic current. This type of process is defined as a faradaic process, and the charges that are passed follows Faraday’s law of electrolysis [39,40]:

(1) Q=mnF.

where *m* is the number of moles of chemical change resulting from the transfer of *Q* coulomb of charge at the interface; *n* is the number of electrons involved in the reaction, and *F* is the Faraday constant with a value of 96,487 $C\cdot {\mathrm{mol}}^{-1}$.

#### 3.1.2. Non-Faradaic Process Current

When an ideally polarizable electrode is immersed into the solution, no charge can transfer across the conductor-solution interface. Therefore, the interface behaves as a capacitor, with one plate of the capacitor being the electrode surface, and the other plate consisting of solvated ions in the solution. The charge at the electrode surface, $q^{M}$, should be equal to that of the solution, $q^{S}$, and is represented by:

(2) $$q^{M}=q^{S}.$$

**Figure 4:**
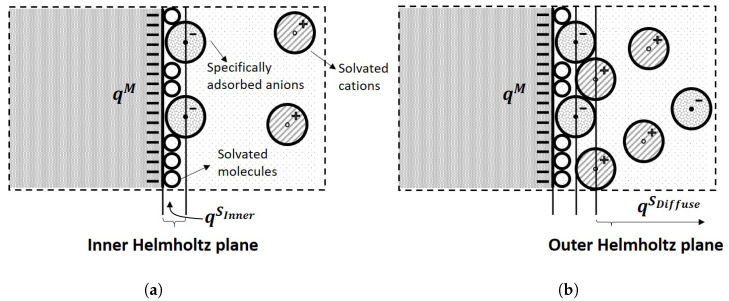
The Inner and Outer Helmholtz Planes: (**a**) The Inner Helmholtz plane consists of physically adsorbed anions and molecular; (**b**) The Outer Helmholtz plane consists of solvent ions due to the long-range electrostatic forces.

When an electric potential is applied to the electrode with respect to the solution, two layers of polarized ions in the solution will accumulate near the electrode surface [41,42] known as the electric double layer, as is shown in Figure 4a.

**Figure 5:**
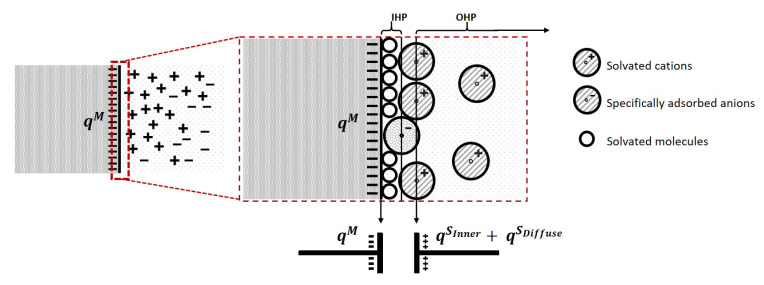
The equivalent capacitor consists of one plate with a electrode surface and the other plate with double layers of inner Helmholtz plane and outer Helmholtz plane at the conductor-solution interface. Specifically adsorbed molecules or ions (anions in this case) are acting as the dielectric, which no charge is permitted to transfer across.

The locus of the centres of nearest specifically adsorbed ions is called the Inner Helmholtz Plane (IHP). These charges are not able to transfer across the inner Helmholtz plane, and therefore, it separates the oppositely polarized ions in the solution from the electrode surface, behaving as a molecular dielectric in a capacitor. The second layer is built of solvent ions that are redistributed due to the long-range electrostatic forces. The locus of the centres of those ions is called the Outer Helmholtz Plane (OHP), which is acting as the other plate of the capacitor, as is shown in Figure 4b. This type of adsorption is said to be nonspecifically adsorption. The region where the ions are redistributed due to the nonspecific adsorption is called the diffuse layer, which extends from the outer Helmholtz plane into the bulk of the solution, as is shown in Figure 5.

The resulting capacitor formed by these two layers is called the double layer capacitor, as illustrated in Figure 5. The charge at the inner Helmholtz plane and outer Helmholtz plane should oppositely match with the charge of the electrode, which is given by:

(3) $$q^{M}=\;q^{S_{inner}}{+\;q}^{S_{diffuse}}.$$

where $q^{S_{inner}}$ is the charge at the inner Helmholtz plane and ${\;q}^{S_{diffuse}}$ is the charge at the diffuse layer.

A double layer capacitor is not an ideal capacitor, which means its capacitance is not constant like a typical parallel plate capacitor. Typically, the value of the double-layer capacitor is a function of the applied potential, with a range of 10 to 40 $\mu {F/\mathrm{cm}}^{2}$ [43]. The current passing through the double layer capacitor when a potential is applied refers to a non-faradaic process current.

### 3.2. Equivalent Circuit of Voltammetric

**Figure 6:**
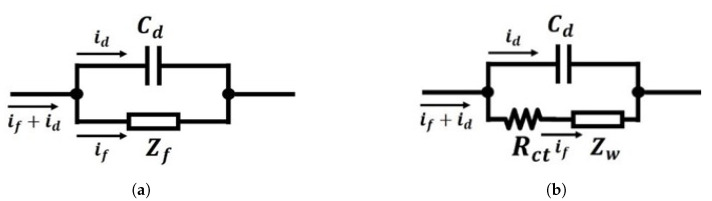
The equivalent model of an electrode conductor-solution interface: (**a**) an equivalent model with a capacitor and faradaic impedance in parallel; (**b**) equivalent model with faradaic impedance represented by a charge transfer resister and the Warburg Impedance.

An electrochemical cell or a voltammetric cell can be considered intuitively as an impedance when excited by a small sinusoidal signal, so its performance should be able to be modelled as an equivalent electronic circuit consisting of capacitors and resistors. As is discussed in Section 3.1, the current flowing through the electrode with an applied potential consists of two types of currents: The faradaic process current, $i_{f}$, and the double-layer capacitor charging current, $i_{d}$, therefore, the structure of the equivalent circuit at the conductor-solution interface should include a capacitor and an impedance in parallel, as is shown in Figure 6a.

The double-layer capacitor $C_{d}$, in Figure 6a experimentally behaves as a real capacitor and $Z_{f}$ represents the faradaic impedance, which is related to the rate of charge transfer reactions at and near the electrode-solution interface. The faradaic impedance can be regarded as a combination of a charge-transfer resistance $R_{ct}$ and an impedance $Z_{w}$ denoted as the Warburg Impedance [44,45,46]. The Warburg Impedance describes the mass transport of the electroactive species, as is shown in Figure 6b.

When the electric potential at an electrode is at equilibrium the parameters of the faradaic impedance can be calculated via [47]:

(4) $$Z_{f}=R_{ct}+Z_{w}=R_{ct}+\frac{\sigma }{\sqrt{\omega }}-j\frac{\sigma }{\sqrt{\omega }}.$$

Therefore, the Warburg Impedance is given by:

(5) $$Z_{w}=\frac{\sigma }{\sqrt{\omega }}-j\frac{\sigma }{\sqrt{\omega }}.$$

where

(6) $$\sigma =\frac{R\;T}{\sqrt{2}{\left(n\;F\right)}^{2}\;A}\left[\frac{1}{\sqrt{D_{O}}\;C_{O}^{*}}+\frac{1}{\sqrt{D_{R}}\;C_{R}^{*}}\right],$$

and the charge transfer resistance is:

(7) $$R_{ct}=\frac{RT}{nFC_{O}^{*}}.$$

where, *R* is the universal gas constant (8.314 J/mol·K),

*T* is the temperature (*K*),

*F* is the Faraday constant (96,485 $C\cdot {\mathrm{mol}}^{-1}$),

*n* is the electron transfer number of the reaction,

$C_{O}^{*}$ is the initial concentration or bulk concentration of the oxidant,

$C_{R}^{*}$ is the initial concentration or bulk concentration of the reductant,

$D_{O}$ and $D_{R}$ are the diffusion coefficiencies of the reactants Ox and Rd,

*A* is the reaction area,

ω is the excitation signal angular frequency,

$\beta_{O}$, $\beta_{R}$ and σ are kinetic parameters of $Z_{f}$.

The double-layer capacitor can be calculated from its inner layer using the equation [48,49]:

(8) $$C=\varepsilon_{0}\cdot \varepsilon_{R}\frac{S}{D}.$$

where, $\varepsilon_{0}$ is the absolute electric constant (8.854 $\times \;12^{-12}\;F\cdot m^{-1})$, $\varepsilon_{R}$ is the relative dielectric constant of the interface, *S* is the surface area of the electrode ($m^{2}$), and *D* is the separation between the electrode plates (m).

**Figure 7:**
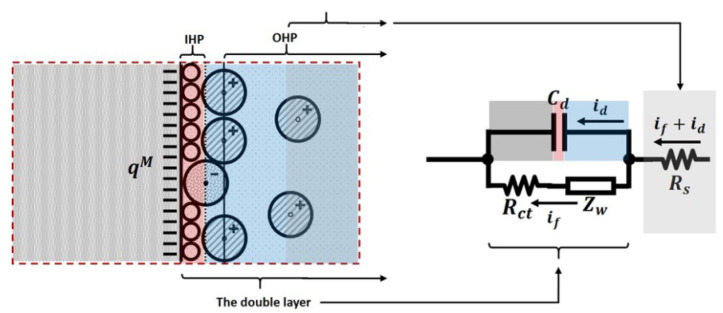
The equivalent model of an electrode conductor-solution interface with solution resistor $R_{s}$.

In addition to the electrode solution impedance, the solution has an associated resistance in the presence of an electric current. Hence, the equivalent circuit presented in Figure 6b, evolves to the equivalent model shown in Figure 7 when the solution resistance, $R_{s}$, is taken into consideration.

Some studies have shown that the solution resistance, $R_{s}$, is related to the ionic concentration, type of ions, temperature, and the geometry of the solution [50,51]. If the bounded area of the solution is *A*, and a uniform amount of current is carried whose length is *l*, where ρ, is the solution resistivity, then [52]:

(9) $$R_{s}=\frac{\rho \cdot l}{A}.$$

**Figure 8:**
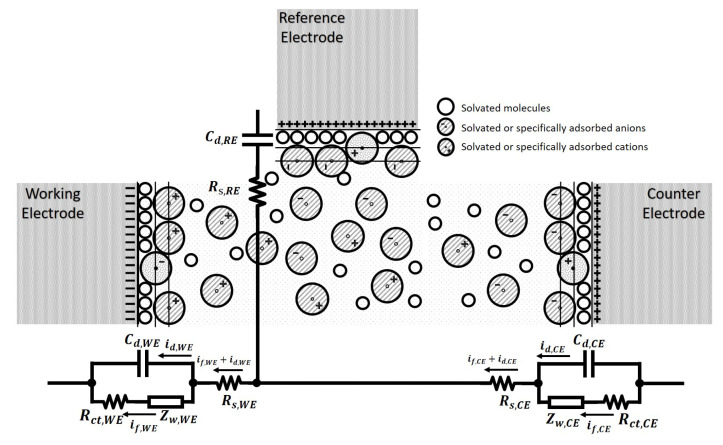
The equivalent electrical model of a three-electrode electrochemical sensor, with the comparison of sensor interfaces of each type of electrodes.

For a three-electrode electrochemical cell, normally the oxidation reaction and reduction reaction occur in pairs, while typically only one of these reactions is of interest, i.e., the one that occurs at the working electrode, WE. The other half of the reaction takes place at the counter electrode, CE. As is mentioned in Section 3.1.1, a reference electrode, RE, is introduced to provide a reference, but will not get involved in redox reactions when measuring the potential difference between the working electrode and reference electrode, as no electrons cross the conductor-solution interface at the reference electrode. Therefore, the structure of a three-electrode reaction cell as shown in Figure 8, where the proposed equivalent circuit of the working electrode and counter electrode are symmetric to reflect the symmetry of oxidation and reduction reactions. As there will be no faradaic current flowing through the conductor-solution interface, and consequently, the model at RE can be simplified to the associated double-layer capacitor and the solution resistance in series, as illustrated in Figure 8.

**Figure 9:**
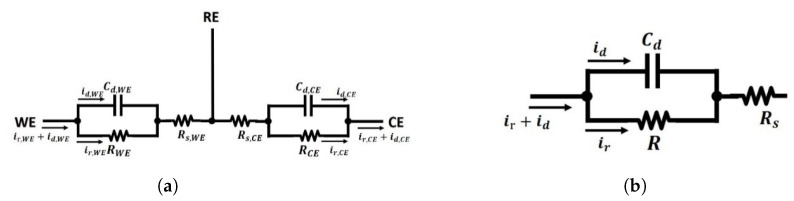
The proposed equivalent electrical model for electrochemical cells. (**a**) The simple equivalent electrical model for a three-electrode electrochemical cell; (**b**) The Randles Model with simplified faradaic impedance to a resistance.

In this model, the Warburg Impedance is included for both the WE and CE as they have complementary chemical reactions at the respective conductor-solution interfaces to maintain the charge within the solution. The Warburg Impedance varies as a function of the applied electrical potential perturbation frequency. At high frequencies, $Z_{W}$ is small since diffusing reactants don’t have to move very far. At low frequencies, the reactants have to diffuse further into the solution, increasing the resultant Warburg Impedance. However, for nanosensor-based electrochemical integrated systems, the mass transport increases, and thus the current is no longer limited by the diffusion of the redox ions toward the WE [53]. As a result, the mass transfer dominated $Z_{w}$ becomes negligible and the model can be simplified to a simple RC circuit as shown in Figure 9a. In addition, as there is negligible current flowing through the RE. Its associated solution resistance and double-layer capacitor can be removed from the equivalent model.

The proposed equivalent electrical model in Figure 9a provide a model for the three-electrode nanosensor electrochemical sensor that reflects the reactions at both the WE and CE. Similarly, it demonstrates the significance of the RE electrode, while also providing an electrical model that can be used for the design of an electrochemical nanosensor-based integrated system [12,22,31,33,34,35,36,45,54]. The model illustrated in Figure 9b, which is part of the general model, is the famous Randles Model [55]. With different considerations of the chemical process, various types of modified Randles models are proposed in order to meet different research targets [47,56,57,58].

## 4. Application of Model

### 4.1. Electrochemical Impedance Spectroscopy

From 1880 to about 1900, the Electrochemical Impedance Spectroscopy (EIS) technique began to establish itself through the work of Oliver Heaviside [59] and now has become an established technique to analyse an electrochemical reaction cell. The electrochemical cell can be regarded as a pseudo-linear time-invariant system, and as such has the following properties:

- An input, $x\left(t\right)$, is mapped to an output, $y\left(t\right)$, and if a scaled input, $a\times x\left(t\right)$, is applied to the system, then a scaled output, $a\times y\left(t\right)$, results;
- If an input $x_{1}\left(t\right)$ is mapped to an output, $y_{1}\left(t\right)$, and, $x_{2}\left(t\right)$ is mapped to $y_{2}\left(t\right)$, then the input $x_{1}\left(t\right)+x_{2}\left(t\right)$ is mapped to $y_{1}\left(t\right)+y_{2}\left(t\right)$;
- If an input x(t) is mapped to an output y(t), and if there is a delay *T* at the input, x(t−T), then the same delay will be resulted in the output, y(t−T).

In fact, an electrochemical cell doesn’t behave like an electrical circuit [43], and it is only considered as a pseudo linear time-invariant system.

In order to study the impedance of an electrochemical cell, a small excitation signal, normally, a small amplitude sinusoidal signal, $V_{t}=V_{0}\cdot sin\left(\omega t\right)$, will be used as the input, where ω indicates the angular frequency of the excitation signal, $V_{0}$ is the peak amplitude and t represents time. Then the resultant current, $I_{t}$, will be a function of a phase shift, ϕ, and the applied frequency and is given by $I_{t}=I_{0}\cdot sin\left(\omega t+\phi \right)$, where $I_{0}$ is the peak amplitude of the resultant current.

Therefore, the resultant impedance of the cell is determined by:

(10) $$Z_{cell}=\frac{V_{t}}{I_{t}}=\frac{V_{0}\cdot sin\left(\omega t\right)}{I_{0}\cdot sin\left(\omega t+\phi \right)}=Z_{0}\cdot \frac{sin\left(\omega t\right)}{sin\left(\omega t+\phi \right)}.$$

where $Z_{cell}$ is the impedance that can be expressed in terms of a magnitude $Z_{0}$ and a phase shift ϕ.

With Euler’s relationship, the impedance of the cell, $Z_{cell}$, can be written as:

(11) $$Z{\left(\omega \right)}_{cell}=Z_{0}\cdot \frac{e^{j\omega }}{e^{j\omega +\phi }}=Z_{0}\cdot e^{-j\phi }=Z_{0}\cdot \left(cos\phi -jsin\phi \right).$$

**Figure 10:**
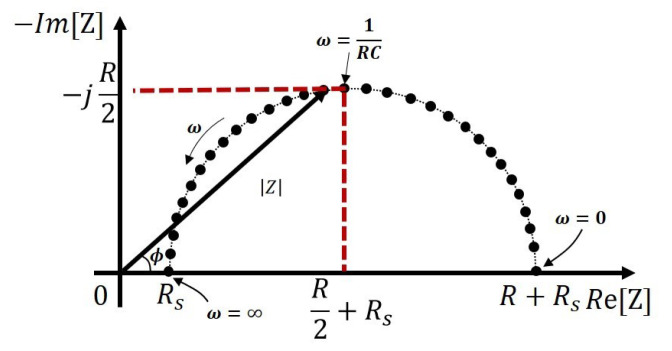
The Nyquist Plot of the Randles Model with an applied AC input excitation.

If the real part is plotted on the X-axis, and the imaginary part is plotted on the Y-axis of a chart, then this is called a Nyquist Plot. The Nyquist Plot for Randles Model illustrated in Figure 9b is shown in Figure 10.

The impedance of the Randles Model shown in Figure 9b:

(12) $$Z_{cell}=R_{s}+\frac{R}{1+j\omega RC_{dl}}.$$

From the Nyquist Plot illustrated in Figure 10, it can be seen that when ω→0, i.e., the applied excitation signal is a DC signal, then the impedance is given by $Z_{cell}=R_{s}+R$, while if ω→∞, then the impedance simplifies to $Z_{cell}=R_{s}$. When $\omega =\frac{1}{RC_{dl}}$, then the impedance now reduces to $Z_{cell}=R_{s}+\frac{R}{2}-j\frac{R}{2}$. With a known R, the double-layer capacitor is be given by $C_{dl}=\frac{1}{\omega R}$.

**Figure 11:**
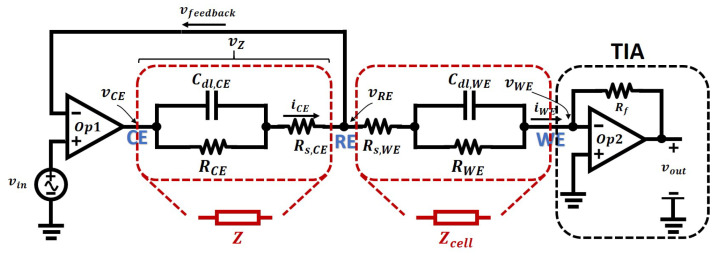
The simplest configuration of a three-electrode sensor equivalent circuit with a potentiostat for simulation.

Therefore, EIS contributes to identifying and characterizing the parameters of the equivalent electrical model of an electrochemical cell, particularly, the double layer capacitor, solution resistor, and the resistance due to the charge transfer resistor. The Randles Model can be used to describe the behaviour of a two-electrode electrochemical cell, and by applying a sinusoidal excitation signal, the impedance of the cell is easy to measure. However, for a three-electrode electrochemical sensor, Randles Model no longer reflects the complexity of the sensor. As demonstrated by the model shown in Figure 9a, the cell voltage will be applied across the working and counter electrodes, and the potential of the reference electrode will be used to sense the actual electrical potential across the electrode solution interface of the working electrode. The resulting electrical interface, usually called a potentiostat previously discussed in Section 2, is illustrated in Figure 11 below.

The operation of the potentiostat is briefly described here. The electrical potential at RE, denoted as $v_{RE}$, and the applied input signal, $v_{in}$, have the relationship shown in the equation below:

(13) $$\left(v_{in}-v_{RE}\right)\cdot A_{op1}-v_{Z}=v_{RE}$$

where $A_{op1}$ is the open-loop gain of the amplifier, Op1, and $v_{Z}$ is the electrical potential drop across the cell impedance *Z*.

Then the potential at RE is given by:

(14) $$v_{RE}=\frac{v_{in}-\frac{v_{Z}}{A_{op1}}}{1+\frac{1}{A_{op1}}}.$$

As the open-loop gain of an amplifier is tremendously large [60], therefore, the potential at RE is given by assuming $A_{op1}\;\to \infty$, simplifying the previous equation to:

(15) $$v_{RE}\approx v_{in}.$$

The electric potential at the RE is adjusted by the applied potential at the CE, and it is forced to be the same as the applied input signal $v_{in}$. This mechanism is called “virtual shorted”, meaning that the potential at inverting input and non-inverting inputs of an amplifier is the same, due to its feedback configuration and the high open-loop gain of the amplifier.

Furthermore, the extremely high DC input impedance of the amplifier ensures that there will be no current flowing through the feedback path. Therefore, the current at the working electrode is:

(16) $$i_{WE}\approx i_{CE}.$$

The second amplifier, Op2, is configured as a trans-impedance amplifier (TIA), which is used to convert the current, $i_{WE}$, into a voltage so that can be conveniently converted to a digital signal. With negative feedback, the inverting input of Op2, i.e., WE, is maintained at the ground, and the potential across RE and WE is equal to $v_{RE}$:

(17) $$v_{RE}-v_{WE}=v_{RE}-0\approx v_{in},$$

And the potential at RE is given by:

(18) $$v_{RE}\approx v_{in}.$$

The output of Op2 is determined by $i_{WE}$ and $R_{f}$, and its value is given by:

(19) $$v_{out}=\frac{i_{WE}R_{f}\cdot A_{op2}}{1-A_{op2}}\approx i_{WE}R_{f}$$

where $A_{op2}$ is the open-loop gain of the amplifier, and $R_{f}$ is the feedback resistance.

Therefore, for EIS analysis, the potential across RE and WE, and the current flowing between RE and WE are all known, then the impedance of the cell can be experimentally determined by:

(20) $$Z_{cell}=\frac{v_{RE}-v_{WE}}{i_{WE}}=\frac{v_{in}}{i_{WE}}.$$

The parameters of the cell impedance can be identified by comparison with the equation on the Nyquist Plot in Figure 10.

(21) $$Z_{cell}=R_{s,WE}+\frac{R_{WE}}{1+j\omega R_{WE}C_{dl}}.$$

**Figure 12:**
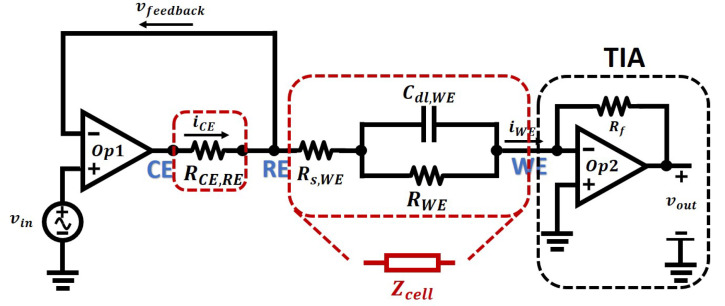
The equivalent circuit with potentiostat for an electrochemical sensor with a simplified structure between CE and RE.

Compared to the simple Randles Model, this three-electrode has the following features:

1 This model is an extension of the Randles model. It models the behaviours of a three-electrode electrochemical sensor, while the Randles model is limited to two-electrode sensors;
2 Randles model will not work well on matching the results between simulation and measurement, as in fact, the electric potential at the working electrode will be hard to control. This three-electrode model controls the potential and measures the current in separated parts of the cell so that the behaviours of the model when doing electrical simulations is an accurate representation of a real electrochemical nanosensor;
3 The impedance *Z* between CE and RE is not critical due to the negative feedback and high open-loop gain of the amplifier, therefore it can be simplified to a resistor in many cases. Then the three-electrode model can be simplified to a Randles model and a resistor in series, as is shown in Figure 12;

### 4.2. The Impact of Solution Resistance for Sensor Electrodes Design

When applying the EIS analysis technique to an electrochemical cell, the components of the faradaic impedance, particularly, the charge transfer resistance, and the double-layer capacitor are of great significance. However, the resistance caused by the solution and slow ion diffusion is no more than an unwanted distraction [38].

**Figure 13:**
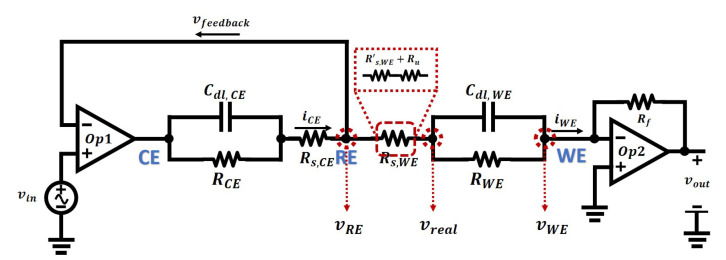
The compensated resistance $R_{u}$ is included within the solution resistance $R_{s}$.

As is shown in Figure 13 above, the actual potential electrical difference at the WE electrode solution interface if given by $v_{real}-v_{WE}$, that is:

(22) $$v_{real}-v_{WE}=v_{RE}-v_{WE}-i_{cell}R_{s,WE}^{\prime }-i_{cell}R_{u}.$$

As the WE is maintained at virtual grounded by the TIA, that is $v_{WE}=0$, therefore the actual electrical potential across the WE electrode solution interface is given by:

(23) $$v_{real}=v_{RE}-\left(i_{cell}R_{s,WE}^{\prime }+i_{cell}R_{u}\right).$$

where $i_{cell}=i_{CE}=i_{WE}$, which is the cell current, and $v_{cell}$ is the electrochemical cell potential that is wanted, and $R_{u}$ is the uncompensated resistance, whose value can be defined as [43]

(24) $$R_{u}=\frac{1}{4\pi \kappa r_{0}}\left(\frac{x}{x+r_{0}}\right).$$

where κ is the solution conductivity, and $r_{0}$ is the radius of the electrode, if it is a spherical electrode, *x* is the distance between the reference capillary tip and the working electrode.

It can be seen that even though the reference electrode tip is just one radius away ($x=r_{0}$), the uncompensated resistance still cannot be eliminated.

In order to minimise the impact of the unwanted solution resistance, one of the approaches is to minimise the current flowing between CE and WE by using nano-meter technology when designing the sensor. In this way, the electrode dimensions can be small values (less than 1 μm) and the typical current flowing through it is only $10^{-15}\;10^{-12}A$ in magnitude. Therefore, the potential drop due to the solution resistance can be neglected, even though the resistance can be significant. However, the surface area of the CE should be larger than that of the WE, in order to ensure that there are sufficient electrons provided by the CE for the chemical reactions at the WE. In other words, the principal chemical reaction should be the electrode solution interface at the WE and should not be limited by the ability of the CE to source or sink electrons

Another method that is commonly utilised to reduce the solution resistance is to decrease the path length between the reference electrode and the conductor-solution interface of the working electrode. In order to achieve this, a Luggin-Haber capillary technique is applied [61], which dramatically makes the reference probe close to the surface of the working electrode.

## 5. Discussion

### 5.1. Double Layer Capacitor and Scan Rates of Cyclic Voltammetry

**Figure 14:**
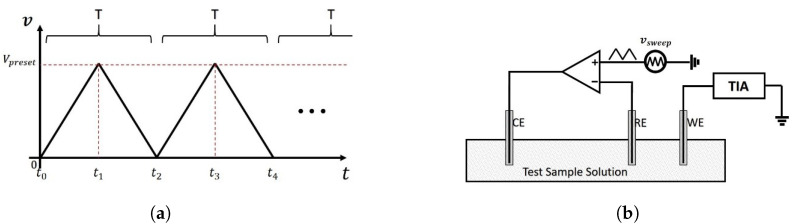
Cyclic Voltammetry. (**a**) the linear sweep signal applied across RE and WE; (**b**) an indicated diagram for CV.

Cyclic Voltammetry (CV) is an electrochemical technique that is commonly employed to study the oxidation and reduction processes of the species under investigation [62]. Typically, like EIS, CV utilises a three-electrode electrochemical cell as well. To perform the measurement, a potentiostat is used to linearly sweep the potential across the reference electrode and working electrode. When the potential reaches a preset limit $V_{preset}$, where point it sweeps back in the opposite direction, and this process is repeatedly performed by triangle signal source, as is shown in Figure 14a. Then the changing current flowing through the working electrode will be recorded in real-time. An indicated diagram for CV measurement setup is shown in Figure 14b. Compared to the one of EIS, the only difference is that the voltage source is not a sinusoidal signal source and instead is replaced with a triangular wave source.

**Figure 15:**
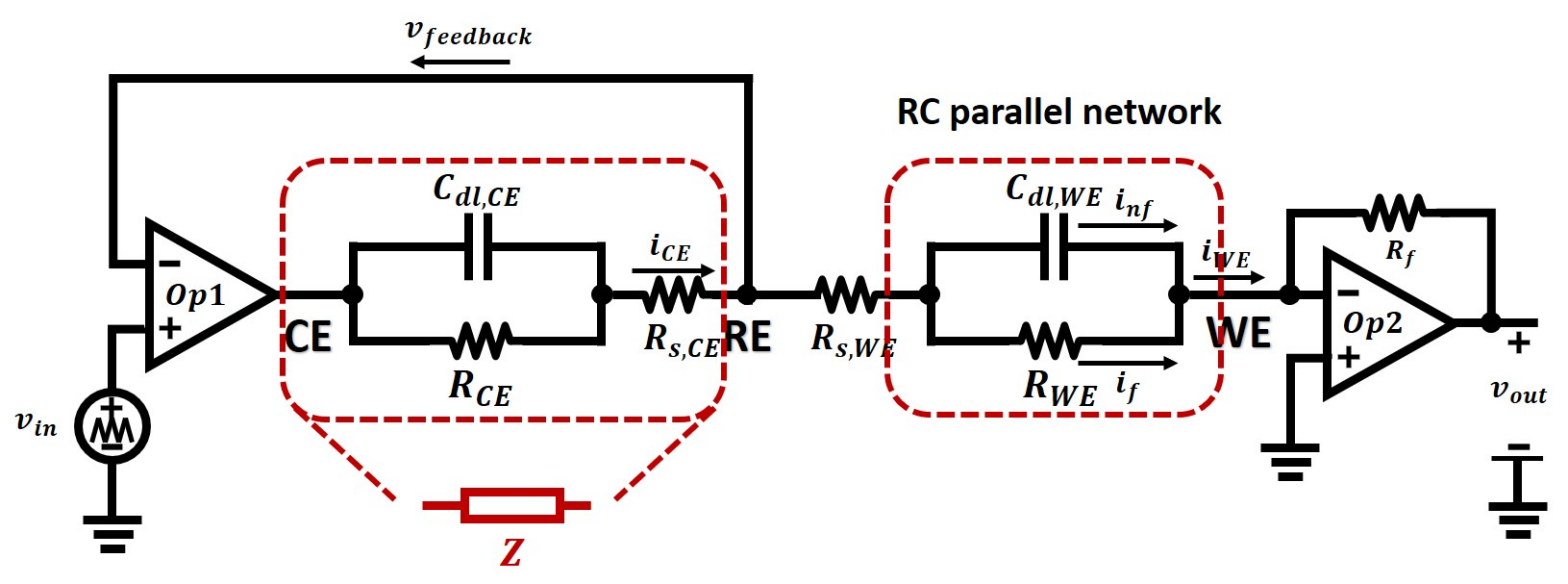
The schematic of CV measurement with a three-electrode sensor model with the absence of Warburg Impedance.

The schematic of the CV measurement setup with a three-electrode sensor model is shown in Figure 15, the potential at the RE is adjusted by changes in the applied potential at CE. This is achieved by the negative feedback configuration of the operational amplifier. The potential at the WE is virtually grounded by the TIA, which is used to convert the current into voltage. Therefore, the change of potential across the RE and the WE $v_{RE}-v_{WE}$ equals to the change of $v_{RE}$. The rate of voltage change over time during a time $t_{1}-t_{0}$ is defined as the experiment’s scan rate, whose unit is V/s. It is calculated by:

(25) $$scan\;rate=\frac{V_{preset}}{t_{1}-t_{0}}.$$

As is shown in Figure 14a, the scan rate can be converted to an angular frequency by:

(26) $$\omega =2\pi \cdot \frac{1}{T}=\pi \cdot \frac{scan\;rate}{V_{preset}}$$

where $T=2\times \left(t_{1}-t_{0}\right)$, is the period of one cycle.

It can be seen that the frequency of the applied signal is proportional to the scan rate. Therefore, the scan rate of the source signal of CV is sometimes described as the frequency of the source signal of CV.

As is shown in Figure 15, due to the faradaic process and non-faradaic process, the cell current $i_{cell}$ flows through an RC parallel network, whose parameters consist of a double layer capacitor and a charge transfer resistance. The magnitude and the phase of the cell current $i_{cell}$ is dependent on source signal angular frequency ω, and according to the circuit theory, the relation of those is given by:

(27) $$i_{cell}\left(j\omega \right)=i_{f}+i_{nf}=\frac{v_{in}\left(j\omega \right)}{R}+j\omega C_{dl}\cdot v_{in}\left(j\omega \right).$$

where $i_{f}=\frac{v_{in}\left(j\omega \right)}{R}$ is the current produced by faradaic process, and $i_{nf}=j\omega C_{dl}\cdot v_{in}\left(j\omega \right)$ is the current produced by the non-faradaic process, namely, the charging current.

It can be seen that if ω increases, the non-faradaic current will dominate the cell current. However, in order to gain the sensory information of oxidation and reduction processes, the current caused by the non-faradaic process should be avoided, therefore, the frequency of the applied signal should not be too high, Namely, the scan rate of CV should not exceed a limit when the non-faradaic current dominates the cell current.

### 5.2. The Feasibility of the Proposed Model

**Figure 16:**
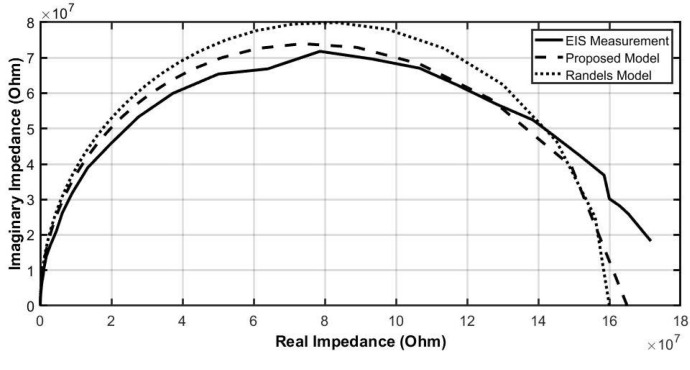
The Nyquist Plot of the models vs. the measurement EIS data.

This model was verified against measured EIS data using our nanowire electrochemical sensors [21], as illustrated. The model parameters were extracted from the measured data ($R_{CE}$ = 35 MOhm, $C_{CE}$ = 20 nF, $R_{s}$ = 8 kOhm, $R_{WE}$ = 130 MOhm, $C_{WE}$ = 1.4 nF) and are consistent with a similar Randles model ($R_{WE}$ = 160 MOhm, $C_{WE}$ = 1 nF, $R_{s}$ = 4 kOhm), as is shown in Figure 16. This demonstrates that our proposed model is applicable in both developing the understanding of electrochemical sensors and model their behaviour in electrical circuits.

## 6. Conclusions

This paper reviews the operation of electrochemical cells and proposes an equivalent electric circuit model for electrochemical nanosensors. The proposed model extends the operation of the well established Randles Model beyond a two-electrode electrochemical sensor to a three-electrode electrochemical sensor. In doing so, the proposed model provides for the first time an equivalent circuit that can for the first time be an EIS based integrated system design. The intuitive nature of the proposed model provides a general sensor design guideline for both engineers and chemists alike, particularly considering the effect of the solution resistance in sensor layout design, control amplifier configurations, as well as the current-readout circuits of the sensors when applying CV or EIS measurements. The proposed model was also extended to include cyclic voltammetry and the resultant interface circuits. A comparison of the two-electrode model and the three-electrode model is presented as well, in order to provide the reference for both engineers and chemists when doing circuits design or measurements. In the end, a fitting simulation is conducted to show the feasibility of the application of three-electrode electrochemical sensors in interface circuits design and electrochemical impedance analysis.

Equivalent impedance models have a promising future in the area of electrochemical nanosensor-based integrated systems, and this paper serves as a guide to both future electronic engineers who will evolve designing sensors or its integrated systems and will provide the supplementary of electronic circuits for and chemists who will conduct electrochemical analysis. 

## Data Availability

The data that support the findings of this study are available from the corresponding author, Ivan O’Connell, upon reasonable request..
